# Diagnosing cystic fibrosis in low- and middle-income countries: challenges and strategies

**DOI:** 10.1186/s13023-024-03506-1

**Published:** 2024-12-20

**Authors:** Michèle Fuhrer, Marco Zampoli, Hugues Abriel

**Affiliations:** 1 Ion Channels and Channelopathies Laboratory, Institute for Biochemistry and Molecular Medicine, University of Bern, Bühlstrasse 28, Bern, CH-3012 Switzerland; 2 Department of Paediatrics and Child Health Division of Paediatric Pulmonology, Red Cross War Memorial Children’s Hospital, University of Cape Town, Rondebosch, Cape Town, South Africa

**Keywords:** Cystic Fibrosis, CFTR, Low- and middle-income countries, diagnostic tools, challenges and strategies

## Abstract

**Background:**

Cystic Fibrosis is caused by recessively inherited variants of the cystic fibrosis transmembrane regulator. It is associated with diverse clinical presentations that can affect the respiratory, digestive, and reproductive systems and inhibit nutrient absorption and growth.

**Main Body:**

The current estimation of people affected by Cystic Fibrosis is likely underestimated as this disease remains undiagnosed in countries with limited diagnostic capacity. Recent evidence indicates that Cystic Fibrosis is more common than initially thought and is likely underreported in low- and middle-income countries. The sweat chloride test remains the gold standard for diagnosing Cystic Fibrosis. However, the costs of commercially available instruments, consumables, and laboratory reagents remain relatively high for widespread implementation in low- and middle-income countries.

**Conclusion:**

Alternative, cost-effective, and simpler approaches to sweat electrolyte measurement, may present more feasible options for CF diagnosis in the setting of low- and middle-income countries. Novel low-cost, point-of-care innovations for measuring sweat chloride should be explored and further validated as suitable alternatives. It will be important to consider how to implement these options and adjust the diagnostic algorithm to meet the needs of low- and middle-income countries. Future Cystic Fibrosis research in low- and middle-income countries should focus on finding a lower-cost and resource-intensive pathway for CF screening and diagnosis to improve its availability.

## Introduction and background

*“Woe is the child who tastes salty from a kiss on the brow*,* for he is cursed and soon must die*.” [1] Fears about the mysterious life-threatening illness of salty sweat, later recognised as cystic fibrosis (CF), or mucoviscidosis, were already prevalent in 15th-century folklore. CF is caused by recessively inherited variants of the cystic fibrosis transmembrane regulator (*CFTR)* human gene on chromosome 7, which encodes the CFTR/ABCC7 anion channel [2]. Pathogenic variants are associated with diverse clinical presentations that can affect the respiratory, digestive, and reproductive systems, and inhibit nutrient absorption and growth [2]. While most CF patients die young, life expectancy has dramatically improved in recent decades [3]. Based on their data registry (2023), the Cystic Fibrosis Foundation (CFF) predicts that the median age of survival for people born between 2019 and 2023 is 61 [4]. Cystic fibrosis is one of the most common autosomal recessive diseases in Europe but also affects individuals of non-European descent [5]. The CFF estimates that 105,000 people of differing ethnicities across 94 countries are diagnosed with CF [4]. This is likely an underestimate as this disease remains undiagnosed in countries with limited diagnostic capacity. The prevalence of CF is thought to be lower in low- and middle-income countries (LMICs) in Asia, Latin America, and Africa, however, the lack of data from these regions suggests that estimates should be taken cautiously [6]. In 2022, 1665 CF patients were reported from nine African countries, most of whom lived in Egypt and South Africa [6]. While upper-middle-income countries such as South Africa and Libya have documented CF in the published literature, 40 other African countries, mainly in the sub-Saharan Region, have no available epidemiological data on the disease [6–8]. Recent studies indicate that CF is more prevalent in Egypt than anticipated [9, 10]. This is likely true in other LMICs across Latin America and Asia, especially in the southeastern region [11–13]. These findings indicate that the CF worldwide burden remains unclear, in large part because LMICs are burdened by more prevalent health issues. Nonetheless, the importance of identifying CF and other rare diseases should not be overlooked, as their impact on affected individuals and families can be profound [11]. 

CF patients in LMICs tend to have a worse prognosis than those from countries in the “Global North,” emphasising the importance of early diagnosis and early access to therapeutic options [12]. While CF diagnosis and treatment have improved in many parts of the world, some regions have not benefited from clinical advancements. This discrepancy underscores an urgent need to address healthcare accessibility, treatment affordability, and diagnostic and therapeutic options in LMICs. Developing effective and cost-efficient CF diagnostics in underresourced areas is an essential first step for improving disease outcomes worldwide.

## CFTR, sweat gland pathophysiology, and clinical manifestations

### The CFTR anion channel

The *CFTR* gene encodes a chloride (Cl^−^) ion channel expressed in epithelial cells of the sweat glands, lungs, pancreas, gut, reproductive systems, and other cell types [14]. The protein is synthesised intracellularly and transported to the plasma membrane, where it controls ion and water secretion [14]. Since its discovery in 1989, knowledge about CFTR structure and function has rapidly expanded. More than 2000 *CFTR* variants with different effects on the protein have been identified, only some of which cause CF-associated symptoms [2, 14]. CF is thus defined as a *genetic channelopathy* [15]. The pathogenic variants are usually grouped into six classes [16]. This includes variants that alter the production (class I) and/or the processing and biochemical maturation (class II) of the protein, regulation of the CFTR channel (class III), Cl^−^ conductance (class IV), reduced quantity (V), and reduced stability (VI) [17]. While carriers of class IV-VI variants usually have milder phenotypes, those with class I-III variants tend to have severe disease [11]. The pathogenic in-frame deletion of phenylalanine 508 (F508del), a class II variant, is the most common CF mutation reported to date, accounting for two-thirds of cases worldwide [18]. Other variants, such as G542X (2.5% allele frequency of all identified variants) and G551D (2.1% allele frequency of all identified variants), are also common [19]. The occurrence of specific variants is strongly linked to the geographic region. While CFTR is a bona fide Cl^−^ channel, it also has an indirect effect on salt absorption by the epithelial sodium channel (ENaC) as well as HCO3^−^, thiocyanate, and glucose transport [13]. 

### Sweat gland physiology

**Fig. 1:**
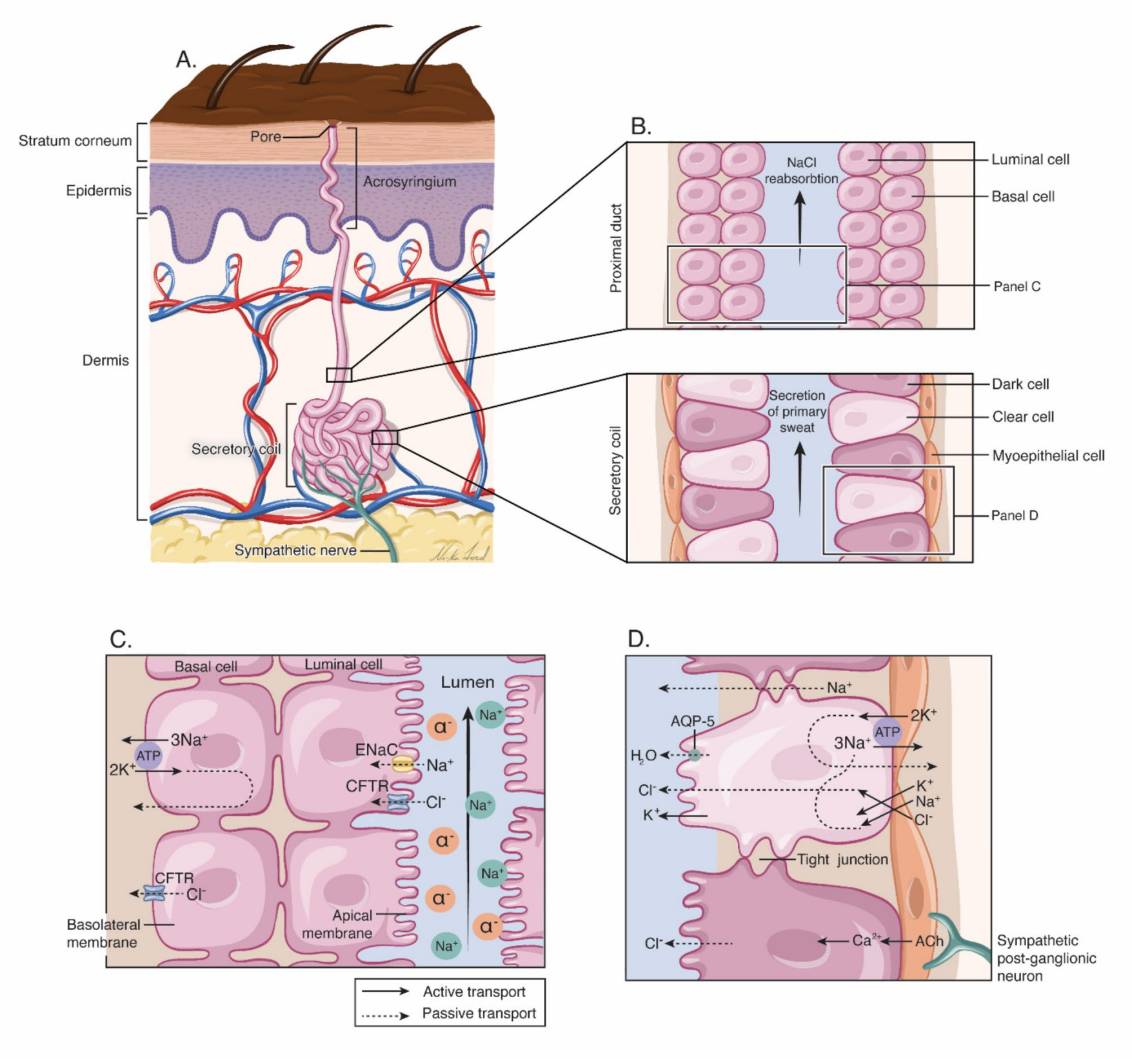
Panels A and B depict the structure of the eccrine sweat gland, including a coiled secretory base and a straight duct leading to the skin. Panel C describes the reabsorption of sodium (Na) and Cl^−^ (Cl) through the CFTR channel in the proximal duct, facilitated by the epithelial Na channel (ENaC) to maintain electrolyte balance. Panel D illustrates the mechanisms of sweat secretion in the secretory coil, which uses acetylcholine (ACh) for stimulation and aquaporin-5 (AQP-5) for water transport. Illustration by Ni-ka Ford, MS, adapted with permission: https://www.ncbi.nlm.nih.gov/pmc/articles/PMC6773238/

CF diagnosis depends on elevated Cl^−^ and sodium (Na^+^) ion concentrations in sweat. Thus, it is worth examining the physiology of sweat glands in more detail. Humans have three types of sweat glands: eccrine, apocrine, and apoeccrine [20]. Eccrine glands are the most common, producing high amounts of sweat, while apocrine and apoeccrine glands have little impact on total sweat production [20]. Sweat glands comprise a secretory coil and a duct leading to the skin (Fig. 1, panel a), and are innervated by cholinergic and β-adrenergic fibres [21, 22]. Both types of fibre are independently stimulated, with cholinergic fibres promoting a greater sweat response than β-adrenergic fibres [13]. Sympathetic nerves innervate the eccrine glands through cholinergic fibres that stimulate muscarinic receptors on the basolateral membrane of epithelial cells (Fig. 1, panel d) [22]. Stimulation of these receptors increases the Ca^2+^ concentration in the cytoplasm of myoepithelial cells, inducing Cl^−^ efflux through Cl^−^ channels in the apical membrane [22]. Sodium can move passively across the cell junction into the lumen due to the electrochemical gradient created by the increased Cl^−^ concentration (Fig. 1, panel d) [22]. Sweat then flows from the secretory coil that produces the primary isotonic sweat into the water-impermeable duct to reach the skin surface [22]. While passing through the duct, sodium and Cl^−^ ions are reabsorbed from the ductal lumen into the luminal cell (Fig. 1, panel c) [22]. The epithelial sodium channel (ENaC) transports sodium through the apical membrane, creating an ion concentration gradient and a potential difference that drives passive Cl^−^ absorption (Fig. 1, Panel c) [22, 23]. The transport of Cl^−^ from the duct lumen into the interstitium occurs through the CFTR [23]. This causes the sweat to become hypotonic and move towards the skin surface.

Cl^−^ ions move in the opposite direction through CFTR channels in the sweat glands than through other CFTR-expressing tissues. Typically, Cl^−^ is transferred from the ductal lumen to sweat gland cells. The secretion of primary sweat still occurs in CF patients, but Cl^−^ is not sufficiently reabsorbed because of a dysfunctional or absent CFTR channel. Sodium is also poorly reabsorbed, increasing its concentration in sweat due to the absence of its coion [23]. Sweat from CF patients is hypertonic and the NaCl concentration is higher than normal sweat, making it an effective diagnostic marker (see below). Notably, the amount of salt lost by sweat is 3–4 times higher in CF patients than in healthy people [24]. Thus, salt replacement therapy is often recommended to patients based on their symptoms, activity, climate, and dietary intake [24]. 

### Clinical manifestations of CFTR variants

**Fig. 2:**
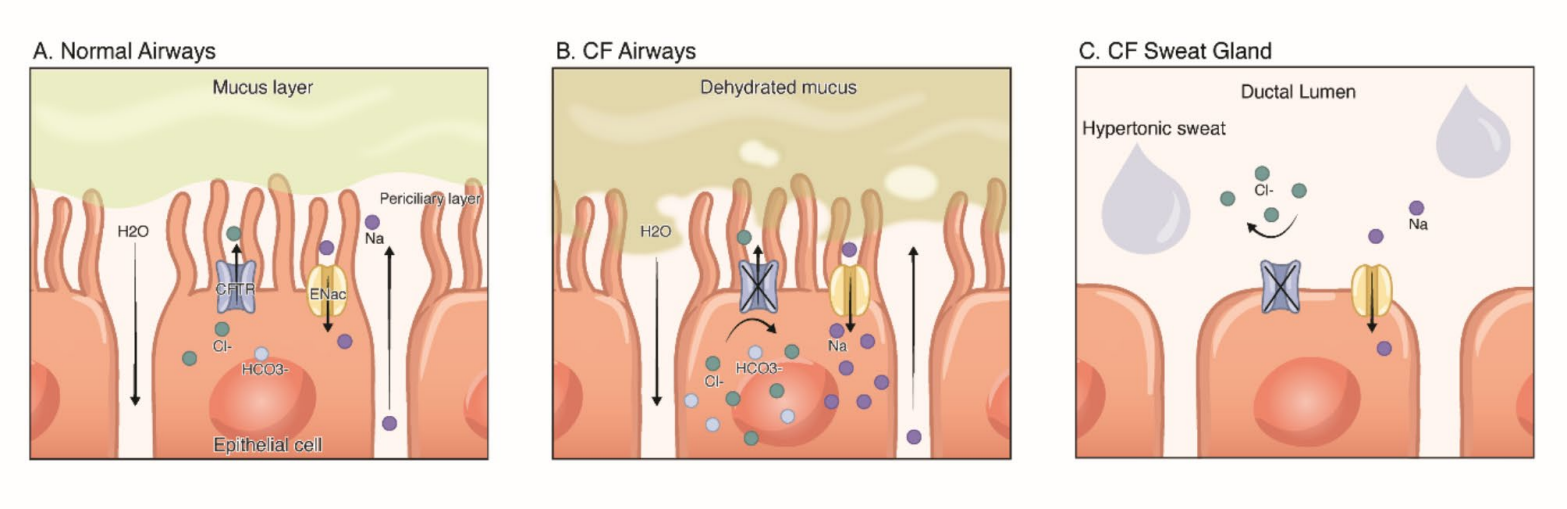
Effects of CFTR on normal airways, CF airways, and CF sweat glands. **A**: CFTR and ENaC work together in healthy airways to optimise airway surface fluid. Cl^-^ and bicarbonate ions pass through the CFTR channel to exit epithelial cells. **B**: In CF airways, Cl^-^ ions and sodium remain in the epithelial cell, and water hyperabsorption occurs. This dehydrates mucus and leads to reduced cilia function, obstructive pathologies, and bacterial colonisation. **C**: The direction of Cl^-^ transport is reversed in the sweat glands compared with other body parts. Cl^-^ ions are not reabsorbed by epithelial cells and remain in the ductal lumen, resulting in hypertonic sweat with relatively high sodium and Cl^-^ concentrations. Illustration by Ni-ka Ford, MS

All known pathogenic variants of the CFTR protein result in reduced Cl^−^ secretion and increased cellular sodium and water resorption in other body parts (Fig. 1) [18]. Increased water resorption in airway epithelia causes the mucus layer to thicken, increasing the risk of obstructions (Fig. 2) [18]. 

Thickened mucus reduces mucociliary clearance in the lung, providing an optimal environment for bacterial infections [18]. Chronic inflammation causes structural damage to the respiratory tract and results in bronchiectasis [18]. The vicious cycle of mucus thickening, reduced clearance, and bronchiectasis leads to ventilation failure and pulmonary insufficiency, the primary cause of death from CF [18]. Obstructions caused by the higher viscosity of secretions can also affect the pancreas. Pancreatic enzymes not effectively released into the intestines can target pancreatic tissue and lead to autodigestion [18]. Pancreatitis or endocrine pancreatic failure that ultimately results in diabetes mellitus can occur [18]. Reduced pancreas bicarbonate secretion can lower the pH, prevent sour stomach chyme from being effectively neutralised or digested, and result in greasy stools, diarrhoea, and nutrient malabsorption [18]. Meanwhile, obstructions in the biliary and hepatic systems can lead to the formation of gallstones, cirrhosis, increased hepatic portal vein pressure, oesophageal varices, splenomegaly, and hypersplenism [18]. 

## Cystic fibrosis in LMICs

### Challenges related to cystic fibrosis diagnosis in LMICs

**Fig. 3:**
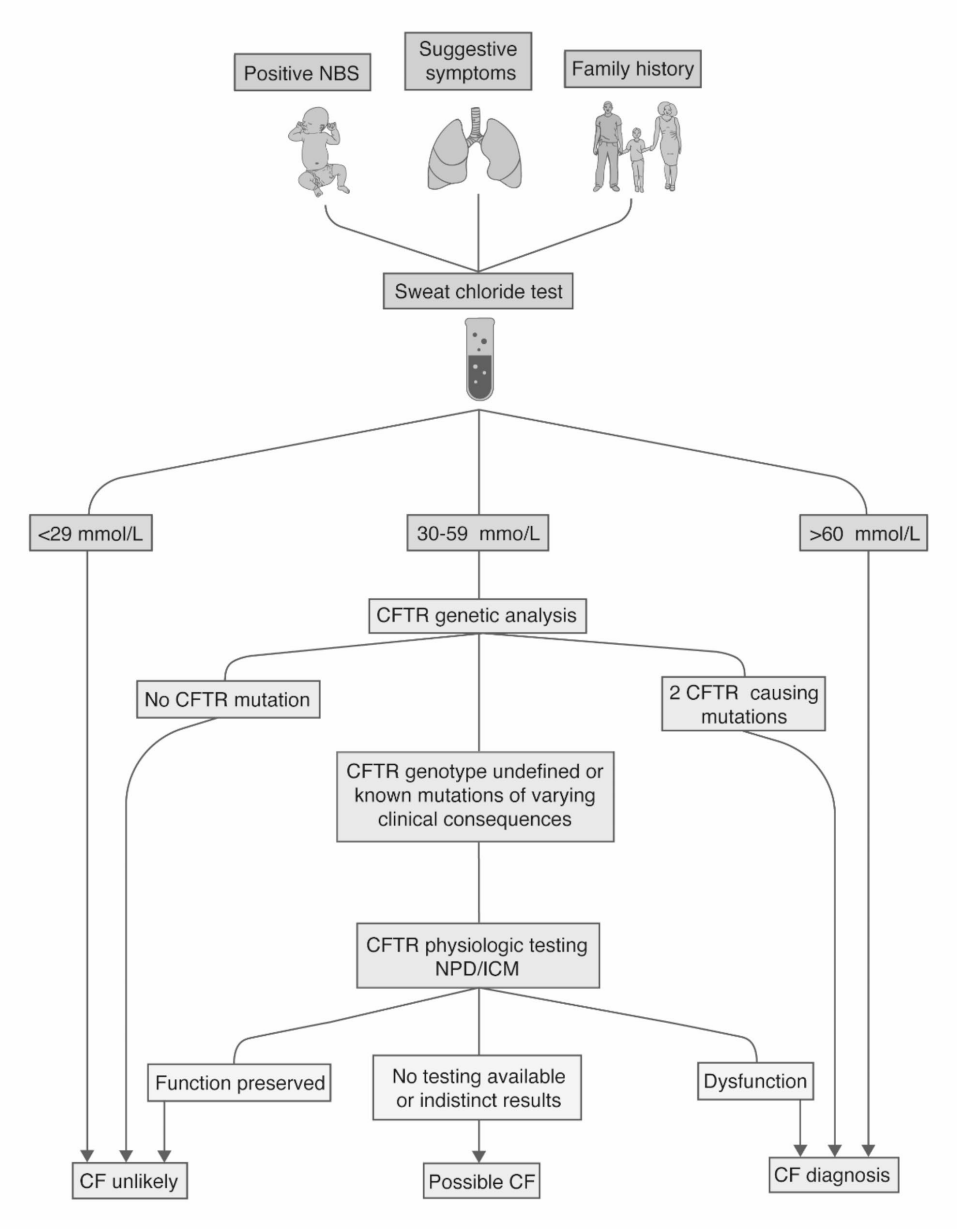
Diagnostic algorithm for CF (consensus guidelines from the CFF, adapted with permission from Farrel et al.) [27]. If the sweat test results are between 30 and 59 mmol/L, the guidelines propose genetic analysis and, if necessary, further testing of the nasal potential difference measurement (NPD) or the intestinal current measure (ICM) [27]. Illustration by Ni-ka Ford, MS

A 2002 World Health Organisation report highlighted reasons why CF is underdiagnosed in LMICs across Africa, Latin America, and India, including lack of knowledge of the disease, restricted access to health care, inaccurate diagnosis, and a high infant mortality rate [25]. In addition, because many LMICs have limited universal newborn screening (NBS) due to resource prioritisation for more pressing healthcare needs, early CF detection is rare. The main objective of NBS is to recognise diseases as early as possible to improve patient outcomes. Different NBS methods and screening tools are used in Europe, the United States, and Australia [26]. Individuals with positive screening results must then undergo further testing to confirm or rule out the disease (see Fig. 3).

The importance of setting up disease registries in LMICs cannot be underestimated. CF registries in high-income countries have informed CF care and promoted research that develops diagnosis and treatment standards. Knowing the prevalence of CF in each country would inform the creation of a network of affected individuals and institutions that care for this patient population. Only a few LMICs, including Brazil, Turkey, and South Africa, have national registries, representing a small percentage of the global CF population [13]. Expanding the number of registries would increase awareness about the global epidemiology of CF.

Research, disease registries, and diagnoses are biased toward particular populations. For example, bias toward the “Global North” has contributed to one-sided medical practices and research approaches. Fatumo et al. found that European populations are overrepresented in genomic studies [28]. In 2021, 86.3% of genomic studies focused on individuals of European descent, 1.1% focused on African individuals, and 0.08% evaluated Hispanic/Latino populations [28]. As a result, knowledge about CF and other genetic disorders remains limited and does not accurately represent population-based differences.

**Fig. 4:**
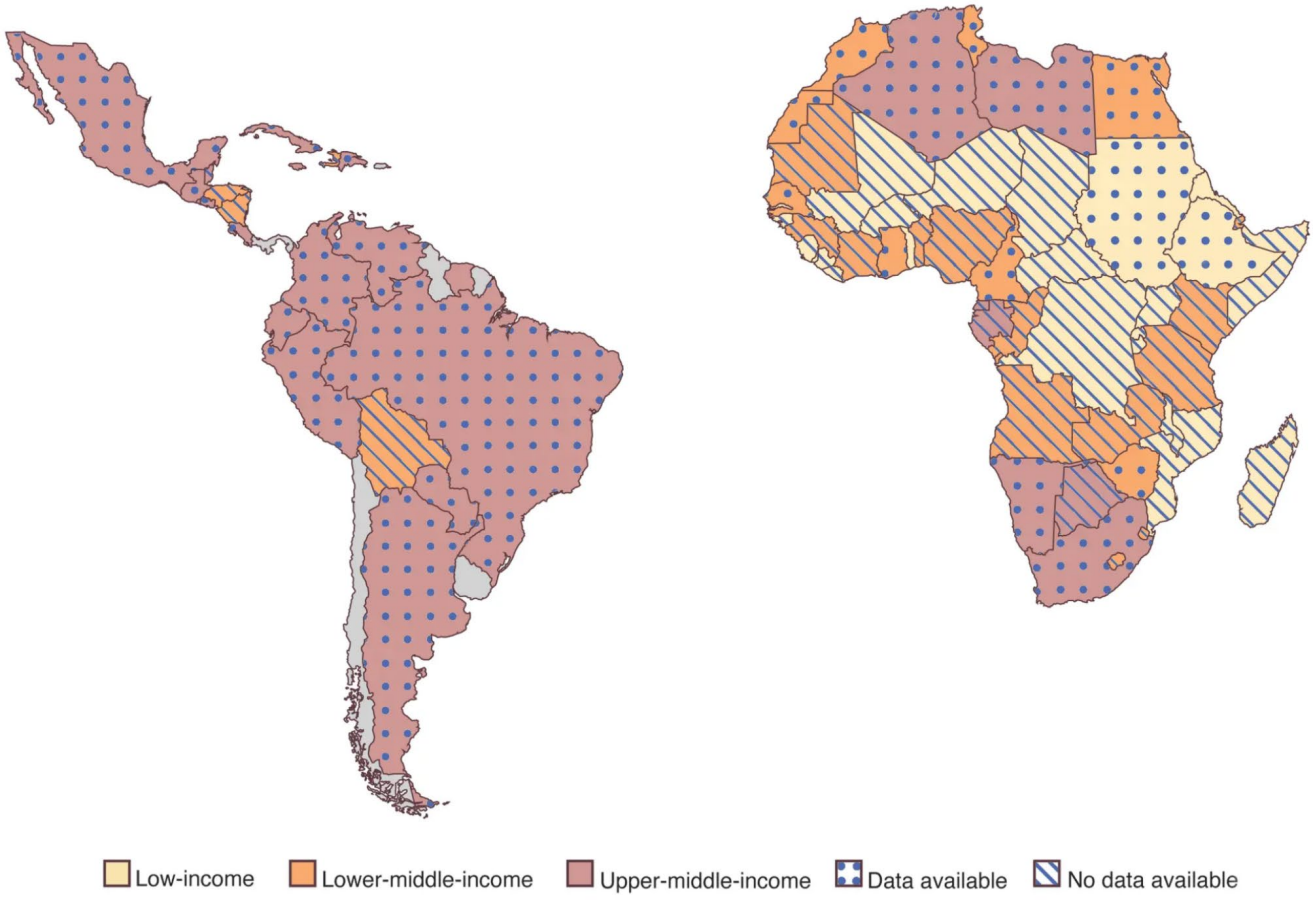
Map of African and Latin American countries by income level (according to the 2023 World Bank report) and CF-related publications [6, 11, 29, 30]. Dotted countries have available data (> 1 publication including patient registries, CF experts, and literature reviews). Striped countries have no published data. Publications involving patients with African or Latin American ancestry who were living in a foreign country were excluded. Illustration by Ni-ka Ford, MS

Figure 4 shows the correlation between economic status and the availability of CF-related data in African and Latin American countries. LMICs, particularly in sub-Saharan Africa, have few publications, highlighting a significant gap in global medical research. CF research tends to be more common in upper-middle-income countries, although there are exceptions at every income level. This disparity underscores the need for more comprehensive studies that encompass the diversity of populations affected by CF and other genetic diseases.

### CF research and diagnosis in Africa

CF is rarely diagnosed in Africa due to a lack of awareness among health professionals, the absence of diagnostic testing capacity, and bias driven by a widespread perception that CF does not impact African populations. In Africa, CF diagnosis is often complicated by the high prevalence of malnutrition and respiratory and gastrointestinal disorders, which can mask or mimic CF symptoms [11, 25]. Insufficient medical care caused by delayed CF diagnosis correlates with a higher rate of disease complications and death [31]. This finding is supported by differences in the life expectancy of European CF patients who receive early diagnosis and adequate therapy and African CF patients who are diagnosed late [31]. A 2021 case report by Owusu et al. highlighted Ghana’s limited resources by describing two children with typical symptoms of CF [32]. Due to the absence of diagnostic tools, their diagnoses were confirmed in South Africa [32].

### CF research and diagnosis in Latin America

CF-related research data and patient quality of care vary significantly across Latin America. General screening initiatives range from the absence of a program in some countries to a fully established national program in others. CF is included in NBS panels in 12 of the 20 countries in Latin America but the extent and effectiveness of these programs differ significantly [33]. Some have *“minimally organised or non-organised NBS activities*,” including Guatemala, Honduras, and the Dominican Republic, while others have “*implemented and launched NBS* activities,” including Ecuador, Peru, and Bolivia [33]. Colombia and El Salvador have NBS but do not screen for CF, while Haiti does not conduct NBS [33]. In a review by Borrajo et al., countries without a working newborn screening program had a higher infant mortality rate than NBS-practising countries, highlighting the importance of early CF detection to assure a better quality of life and a greater life expectancy [33].

The availability of diagnostic tools is unequal over the continent. Sweat and genetic tests are not generally available [34]. Filho et al. noted that greater access to testing would increase the diagnostic rates in Latin America [34]. A higher proportion of diagnosed CF patients would improve the standard of care for affected people.

## CF diagnosis

### Diagnostic algorithm for CF

In 1998, the CFF published a consensus statement with updated guidelines for diagnosing CF that reflected the latest scientific insights and methodologies. This document has been instrumental in standardising CF diagnosis across different healthcare settings [35]. CF diagnosis depends on the following criteria:

- The presence of phenotypic features (chronic sinopulmonary disease, gastrointestinal and nutritional abnormalities, salt loss syndrome, and obstructive azoospermia) [35] OR.
- A history of CF in a sibling OR.
- Positive newborn screening results.

AND.

- Laboratory evidence of a CFTR alteration (shown as elevated sweat Cl^-^ concentration, abnormal nasal epithelial ion transport, or known CF-causing variants in each *CFTR* parental allele) [35].

The clinical evaluation was described as essential, especially for patients with atypical CF symptoms who do not have evidence of a CFTR alteration [35]. The diagnostic criteria for CF have evolved due to advancements in research and a deeper understanding of the disease. As a result, the CFF revised the Consensus Guidelines in both 2008 and 2015 [36, 27]. The definition of CF nowadays still includes the clinical presentation of CF (positive NBS, suggestive symptoms, family history) and evidence of CFTR dysfunction [27]. The new guidelines give a more straightforward structure of the diagnostic process and include a diagnostic algorithm that tests for Cl^−^ levels in sweat (> 60 mmol/L Cl^−^: CF very likely; <30 mmol/L Cl^−^: CF very unlikely; 30–59 mmol/L Cl^−^: more testing is required to confirm CF) [36, 27]. This test is also the primary tool used to confirm the disease [35]. The CFF highlights special patient groups and the challenges of diagnosing CF in some populations, including screened and unscreened patients, NBS and prenatally tested patients, and patients who present clinically but remain unscreened for various reasons (born before the availability of NBS, false negative NBS) (Fig. 3) [36, 27]. Patients with a positive NBS and a sweat test result from < 29 mmol/L or 30 to 59 mmol/L and inconclusive genetic testing are categorized as CFTR-related metabolic syndrome (CRMS), and CF screen-positive, inconclusive diagnosis (CFSPID) (considered to be the same) [27]. Over the past years, expert groups created definitions and additional guidelines for diagnosing special patient groups with conditions associated with CFTR gene mutations, such as CFTR-related disorders (CFTR-RDs) and CRMS/CFSPID [37, 38]. Although patients with these conditions suffer from a CFTR-associated mutation, the diagnostic algorithm does not show a certain CF diagnosis. CFTR-RDs are defined by excluding a CF diagnosis, present clinical features (phenotype), and the evidence of a partially functioning CFTR protein whose lack of activity does not reach CF thresholds [38, 39]. The guidelines recommend performing further tests in a CF clinic after ruling out a non-CFTR cause [38]. After the exclusion of CF, the diagnostic algorithm verifies a CFTR dysfunction in 2 different functional tests (sweat test, nasal potential difference measurement (NPD) or the intestinal current measure (ICM)), OR 1 CFTR variant and dysfunction in at least 2 different functional tests OR 2 CFTR variants with maximum 1 CF causing [38]. CRMS/CFSPID is defined by an infant with a positive NBS result for CF and sweat chloride < 30 mmol/L and 2 CFTR mutations, at least one with an unclear phenotype OR an intermediate sweat chloride 30–59 mmol/L and 0 or 1 CF-causing mutations [40]. For patients with CRMS/CFSPID, guidelines recommend additional genetic testing if < 2 disease-causing variants were found in NBS [37]. Frequent follow-ups should be done with repeated sweat testing until age 8 to detect individuals who may be reclassified to CF and need specific care [37]. Green et al. recommend limiting further laboratory tests such as radiology, microbiology, and pulmonary function testing [37]. 

### Sweat NaCl concentration and sweat testing for CF

While salty sweat has been recognised as a phenomenon for many years, Darling et al. first reported on the higher Cl^−^ concentrations in the sweat of CF patients in 1953 [41]. This informed the development of the landmark sweat test, the quantitative pilocarpine iontophoresis test (QPIT), by Gibson and Cooke in 1959 [42]. Pilocarpine is a naturally occurring alkaloid derived from the *Pilocarpus* plant. Like acetylcholine, pilocarpine acts as a cholinergic agent by stimulating the muscarinic receptor [43]. During iontophoresis, a small electrical current is applied to the patient’s skin to stimulate the transdermal transport of pilocarpine to the eccrine sweat glands and induce sweat secretion [43, 44]. The Gibson-Cooke method uses filter papers to collect the sweat, which is then eluted from the paper before the electrolytes can be chemically analysed [45]. Measuring Cl^−^ levels in sweat is a method approved by CFF guidelines [27]. 

The Gibson-Cooke protocol remained the leading method for testing sweat until Wescor (Elitech/WESCOR, Inc., Logan, UT, USA) introduced novel and more efficient methods of QPIT and sweat collection (Macroduct^®^) in 1983. While Macroduct was the first of its kind, other brands (e.g. Qunatimetrix^®^ Sweat Control for Cystic Fibrosis Testing) now offer similar tests. Macroduct^®^ uses special gel discs containing pilocarpine nitrate (0.5%) and sweat collection in a coiled microtubing collector cup. Collected sweat is then tested for Cl^−^ levels or electrical conductivity using Wescor Sweat-Chek^®^ technology. Sweat conductivity is expressed in mmol/L equivalent NaCl [46]. Wescor later introduced the Nanoduct^®^ device, which stimulates sweat production, collects sweat, and analyzes conductivity using a single machine, eliminating the need to collect a separate sweat sample. Notably, sweat conductivity testing does not represent the actual Cl^−^ or sodium concentrations in a sample. Nevertheless, studies show a strong correlation between conductivity and chloride concentration [47–50]. The conductivity test gives values approximately 15–20 mmol/L higher than the chloride concentration in sweat [51]. This discrepancy arises because other ions, such as bicarbonate and lactate, contribute to the conductive properties of sweat [51]. The Nanoduct^®^ device instructions define a test result of ≥ 80 mmol/L (equivalent NaCl) as abnormal, with further testing required to confirm the diagnosis [46, 52]. 

Regardless of the method, sweat testing requires sweat stimulation, collection, analysis, and result interpretation [23]. Technical or human errors can occur during this process. CFF guidelines emphasise the importance of using experienced and trained staff to ensure correct handling and performance [36]. In 2005, Mishra et al. noted that a well-performed sweat test provides a reliable measure of CFTR function, even in the genomic era [23]. False positive and negative tests are uncommon and are likely caused by underlying conditions, such as hypothyroidism, pancreatitis, malnutrition, untreated adrenal insufficiency, or glycogen storage disease [53]. Since newborns do not produce enough sweat in the first few days of life, the CFF recommends performing the sweat test at ≥10 days of age [4]. A general statement about the costs of the sweat test remains challenging due to its variation from country to country. For example, a sweat test in Switzerland costs approximately CHF 130 ($ 145 in 2021), while the price in Australia is lower at $ 35 (2013) [54, 55]. Not included in this price are the acquisition of the device of several thousand dollars and the cost to train the staff [54]. 

The sweat chloride and sweat conductivity tests show reliable performance characteristics. In a study by Rueegg et al., the Macroduct^®^ shows a sensitivity of 99% and a specificity of 93% [56]. Compared to the Macroduct^®^, the Nanoduct^®^ was shown to be equally sensitive (98%) but with a lower specificity (79%) [56]. Another comparison study of sweat chloride and conductivity from Bedran et al. found similar sensitivity values (98.5%) but better specificity results (99.9%) [57]. Furthermore, the authors showed a PPV of 98.5%, an NPV of 99.9%, and an overall accuracy of 99.8% for sweat conductivity [57]. The Nanoduct^®^ conductivity test is shown to effectively differentiate between CF and non-CF conditions and results correlate well with the original and gold-standard methods [51, 58, 59]. The conductivity results align with the chloride concentration detected using the Gibson-Cooke method [51, 58, 59]. Given this high diagnostic accuracy, the sweat conductivity test has been proposed as a diagnostic tool to supplement screening [47–49]. The diagnostic algorithm was developed for use in high-income nations and does not account for limitations in resources, such as materials and funding, in LMICs.

## Newborn screening

NBS for CF can improve the quality of life and lifespan of affected individuals [26, 60]. Each country must consider testing costs and material resources and use strategies adjusted to its economic circumstances. NBS methods can test for immunoreactive trypsinogen (IRT), pancreatitis-associated protein (PAP), and gene expression. Trypsinogen is the proenzyme for trypsin, a proteolytic enzyme. It is produced by the pancreas and transferred to the intestines, where another enzyme activates it into trypsin, which breaks protein down into smaller pieces for further digestion [61]. The immunoreactive trypsinogen test was developed in the 1970s and is used for selective screening but not for diagnosis. Patients with elevated trypsinogen results/positive NBS results have an increased risk of CF but require further testing by sweat or genetic testing to confirm or exclude the disease [62]. PAP is an acute-phase pancreatic protein that can be elevated in patients with pancreatic stress [63]. Current NBS methods are two-staged, using the IRT as the primary test. Following a positive IRT, a second IRT (IRT/IRT), genetic testing (IRT/DNA), pancreatitis-associated protein testing (IRT/PAP), or even a three-tier test approach is used, depending on the country. Countries using DNA as a second tier constantly expand the NBS panels, resulting in more positive screening results, including carriers of genetic diseases [64]. In 2003, the estimated annual cost of CF NBS in Wisconsin was $4.58 per newborn, and it is described as a cost-saving alternative to traditional diagnosing methods [65]. 

### Challenges of implementing NBS in LMICs

Only a few LMICs have implemented NBS. Gaikwad et al. encouraged all developing countries to develop and integrate a national neonatal screening program into the national healthcare system [66]. In addition to screening for common disorders, NBS programs should test for CF [66]. Implementing NBS in LMICs faces several challenges, including potential financial constraints. Studies from various African countries show that new mothers are discharged from the hospital early (≤24 h after birth) [66–68]. This is problematic since most NBS is performed after this time point. Thus, an adequate length of hospital stay should be encouraged to limit complications in both mothers and children [68]. 

Once a patient is discharged, follow-up is not always performed because the individual is untraceable, relocates, rejects the examination, or dies [69]. Clear communication about the disease and its consequences before discharge is essential for mothers to understand the significance of infant follow-up and further testing. A robust recall system must be established to prevent loss-to-follow-up if an appointment is missed.

Another challenge to NBS is the increase in the number of home births. A third of children are born at home rather than in a clinical setting [70]. In countries in the sub-Saharan zone, the proportion of homebirths is even greater. In Chad, Ethiopia, and Niger, for example, the rates are 78%, 73%, and 70%, respectively [70]. Only 13% of new mothers in this region received postnatal follow-up, which is essential for preventing complications in both mothers and children [71, 72]. Newborns born at home are often not adequately examined or screened. NBS programs should consider implementing postnatal home visits for this patient population.

Given their differences, NBS programs in northern countries such as Europe and the USA cannot be used by LMICs. When creating a new NBS system, it is essential to account for the structural circumstances of the country.

## Alternative diagnostic tools

Novel alternative tools, including nasal electrical potential difference (NPD), intestinal transepithelial current (ICM), and the β-adrenergic sweat test, are available to measure CFTR function. The NPD and ICM tests evaluate CFTR physiology and are useful when genetic or sweat test results are ambiguous (Fig. 3) [27]. The NPD test assesses ion transport across the nasal epithelium and measures differences in the electrical potential. Patients with CF have a more negative potential than healthy individuals due to higher levels of sodium absorption caused by CFTR dysfunction. In its 1998 consensus statement, the CFF accepted the NPD test as a diagnostic tool [35]. The ICM quantifies transepithelial ion current (short circuit current, Isc) changes in a rectal biopsy sample [54]. CFTR function can be assessed by reflecting the transepithelial transport of ions such as Cl^-^, HCO3-, and K +  [54, 73]. Studies have shown NPD testing results with a sensitivity of 94.8–100% and 96.5–100% sensitivity compared to healthy patients [74–76]. ICM applies to be more sensitive compared to NPD [54]. While the NPD costs 600–800 CHF in Switzerland, Fernandez et al. could not give further information about the cost of the ICM test [54]. 

The β-adrenergic sweat secretion test measures the CFTR-mediated secretion of Cl^-^ in the sweat gland, a value proportional to the activity of the CFTR channel. CF patients respond normally to cholinergic-induced sweating but do not secrete sweat when stimulated through the β-adrenergic receptor via cAMP-dependent signaling [77, 78]. Studies achieved a sensitivity and specifity up to 100% in β-adrenergic sweat secretion test compared to the adult control group [79]. Fernandez et al. could not make a statement about the price of the test [54]. 

This test is complex and requires sophisticated infrastructure and special materials. Alternate methods are often used if the sweat test and genetic results are inconclusive, so few patients require β-adrenergic sweat secretion testing. However, because of its complexity, this method may not be suitable as a CF diagnostic in LMICs.

### Genetic testing

CF is an autosomal recessive inherited disease. *CFTR* gene variants can be identical (homozygous) or different (compound heterozygous). The diagnostic algorithm suggests that genetic testing can help confirm a CF diagnosis for individuals with intermediate sweat test results (Fig. 3) [27]. Since the consensus guidelines do not recommend a specific DNA test, laboratories often begin by using commercially available CFTR panels of pathogenic variants, followed by either Sanger or next-generation sequencing (NGS) [80, 81]. To guarantee high-quality care, the European Cystic Fibrosis Society (ECFS) revised its best practice guidelines in 2018 [82]. Castellani et al. established criteria for laboratories that perform DNA analysis to ensure reliable CF diagnoses [82]. The guidelines advise extracting DNA from EDTA blood, a buccal swab, or dried blood spot samples [82]. Test kits should contain a limited panel of the most frequent pathogenic CFTR variants in the region and be able to identify at least one mutated allele in 96% of CF patients [83]. If only one variant is identified, extended gene sequencing should be performed to identify rare variants and molecular abnormalities, such as duplications and deletions [82]. ECFS and CFF guidelines specify that CFTR2 is the reference database for specific CF variants [19, 27, 82]. The website provides variant-specific information and is updated regularly [19]. The ECFS recommends confirming pathogenic variants identified by DNA analysis using the CFTR2 website [82]. If a CFTR defect has not been reported, the novel variant should be submitted to databases to collect information and better understand its potential clinical significance [82]. A CF diagnosis is confirmed if the patient has two variants occurring on separate chromosomes (in trans) and is classified in a relevant database, such as CFTR2 [82]. However, failing to identify two disease-causing variants after DNA analysis does not exclude CF if typical symptoms or abnormal CFTR physiology are present [82]. 

Patients with pathogenic variants that cause CF or CFTR-related disorder (CFTR-RD) should be transferred to an institution with specialised CF care [82]. DNA analysis provides a high level of specificity for CF patients, depending on the panel chosen for the local population. A recent study revealed improvements in the sensitivity and specificity of genetic tests, achieving 98.8% sensitivity and 99.6% specificity in US laboratories [84]. Nevertheless, it is important to note that the predominance of data from populations of European descent skews current knowledge about the spectrum and distribution of CFTR variants. Thus, other panels with geographically specific pathogenic variants should be developed for each region. There is a clear need for further genetic analysis in countries with little or no available data. Genetic testing also provides additional information, such as the suitability of a pathogenic variant for CFTR modulator therapy, that can help inform treatment options. Genetic testing can also be used for carrier screening during family planning. Despite the additional information provided by genetic testing, the CFF advises against making prognostic predictions based on genetic analysis because no direct correlation between genotype and phenotype has been demonstrated [36]. 

Laboratory access, high cost, and the lack of material resources and genetic panels suitable for local populations are limiting factors for the broad use of genetic testing in LMICs. However, this testing should be promoted despite these limitations, since confirming CF and evaluating therapeutic options is essential. It is also likely that new DNA sequencing technologies, such as the long-read-based nanopore approach developed by Oxford Nanopore Technologies, will enable more laboratories in LMICs to perform genetic testing due to their lower investment costs and ease of use (El Makhzen et al. in preparation).

## Future outlook and alternative solutions

New approaches are needed to improve CF diagnostic tools in LMICs. Experimental options such as organoids and β-adrenergic tests are not likely to be achievable in a short time frame. Thus, alternative solutions should be considered for these regions.

### Microneedle patches

Among the possible alternatives are microneedle (MN) patches, which have the potential to optimise existing tools to meet the needs of LMICs. Li et al. used MN patches to stimulate and collect sweat from horses and found that they induced consistent sweat production and had double the sweat collection density as the standard method [85]. The authors concluded that this technique could be a simple and accessible alternative for sweat induction to diagnose CF [85]. Since the manufacturing costs are expected to be <$1 per patch, this technology could be widely used in resource-limited settings [86]. A research team from Atlanta (USA) used MN patches as part of the sweat test and performed the first clinical trial on healthy adults [87]. The patches induced a similar sweat concentration as iontophoresis and higher Cl^−^ levels [87]. Since this is the only human study conducted to date, clinical trials are needed to assess the utility of MN patches in different subpopulations, including individuals suspected of having CF. The higher Cl^−^ level in the sweat induced by these patches should also be evaluated.

### Sweat stickers

Sweat stickers were recently developed as a low-cost method for diagnosing CF patients. Sweat is stimulated through pilocarpine iontophoresis using the Macroduct^®^. The sweat stickers are only a millimetre thick and have an integrated chloride analysis [88]. The epifluidic device (sweat stickers) have an optimised microfluidic design for efficient sweat collection and analysis [88]. The multilayered structure makes the sticker skin compatible and allows it to seal tightly to the epidermis to reduce leakage during sweat collection [88]. The sticker is then removed and the sweat is extracted and analysed [88]. According to Ray et al., stickers collect 33% more sweat than currently used methods [88]. Ray et al. discussed another approach to analysing the sweat collected from a sticker. As Cl^-^ in the sweat reacts with the silver chlorinate agents in the sticker, it produces a colour proportional to the ion concentration [88]. The built-in colorimetric sensors then analyse the Cl^-^ concentration by taking a picture with a smartphone camera [88]. Given the advanced image processing method, there is no need for sweat extraction or external instruments [88]. While this method has only been tested on a few patients, its accuracy matches laboratory-based diagnostics [88]. The recognition of color using optical imaging functions well at high Cl^-^ concentrations but has some problems at lower concentrations, increasing the risk of false negative diagnoses [88]. Further investigations are needed to improve the accuracy of colorimetric smartphone analysis for clinical validity [88]. A wearable electrochemical sensor has the potential to diagnose CF both outside the hospital and in a clinical setting.

### Colorimetric assays

Kim et al. introduced a miniaturised wearable platform for quantitative biomarker analysis [89]. The device combines the stimulation, collection, and biochemical analysis of different sweat components and requires only a few microliters of sweat [89]. An electronic circuit, electrodes, hydrogels with pilocarpine, and colorimetric biosensors are integrated into the device [89]. The platform is attached to the skin, sweat is collected in the microfluidic channels and the micro reservoirs are initiated by activating a switch [89]. Different biomarkers, such as chloride, an indicator of CF, zinc, and iron, are analysed to gain insight into the patient’s nutritional status and detect any deficiencies associated with malabsorption [89]. The colorimetric approach is as reliable and accurate as the macroduct approach and has the potential to be used in regions with limited resources [89]. However, more studies with larger patient groups are needed to define biomarker standards for CF patients.

### Quantum test

The CFQuantumtest (CFQT) was developed to simplify the detection of sweat chloride [90]. The electrode in the quantum test is portable and not attached to an external device [90]. Skin is stimulated to produce sweat through iontophoresis with pilocarpine gel (similar to the Macroduct^®^) [90]. As sweat comes in contact with the chloride test patch, the color undergoes a chemical reaction and changes into a dark red ring which indicates the amount of collected sweat [90]. The Cl^-^ ions form a white precipitate on the patch, with its surface being proportional to the amount of Cl^-^ [90]. A camera and software are then used to evaluate the amount of chloride in the sample [90]. While CFQT can efficiently differentiate between CF patients and healthy individuals, improvements are needed to reduce the number of invalid tests and improve the accuracy of the sweat chloride measurements [90]. While a follow-up study found that CFQT is not yet diagnostically or analytically reliable, its approach may be used to inform future research [91]. 

### Saliva

CF is one of several diseases that can affect the composition and use of saliva as a diagnostic marker [92]. Gonçalves et al. studied the chloride and sodium concentrations in saliva to avoid the challenges of iontophoresis [93]. Saliva sample collection is non-invasive, quick, inexpensive, and easier than sweat testing [93]. In the Gonçalves et al. study, the chloride and sodium concentrations were higher in CF patients than in healthy individuals [93]. It was concluded that saliva could not be used because of the method used to determine the ion concentration [93]. However, the authors highlighted the potential of using saliva as an alternative to sweat testing in the future [93]. Since CF also influences other parameters in saliva, including potassium, pH, and volume, future investigations should focus on defining diagnostic markers and determining the correct method for measuring ion concentration [93]. 

## Conclusion

Recent evidence indicates that CF is more common than initially thought and is likely underreported in LMICs. Without a universal NBS program, the true CF incidence in LMICs will remain unknown, since many undiagnosed infants are likely to continue dying at a young age. Health authorities and practitioners in LMICs should be encouraged to invest more in CF recognition and diagnosis, beginning with increased access to basic diagnostic tools such as sweat testing and DNA analysis. The sweat chloride test remains the gold standard for diagnosing CF. However, the costs of commercially available instruments, consumables, and laboratory reagents remain relatively high for widespread implementation in LMICs. Alternative, cost-effective, and simpler approaches to sweat electrolyte measurement, such as sweat conductivity, may present more feasible options for CF diagnosis in these settings. Novel low-cost, point-of-care innovations for measuring sweat chloride should be explored and further validated as suitable alternatives for LMICs. It will be important to consider how to implement these options and adjust the diagnostic algorithm to meet the needs of LMICs. Future CF research in LMICs should focus on finding a lower-cost and resource-intensive pathway for CF screening and diagnosis to improve its availability.

## Data Availability

All data and information analysed in this study are included in this published article and its references.

## Abbreviations

CF: Cystic fibrosis
CFF: Cystic Fibrosis Foundation
CFTR: Cystic fibrosis transmembrane conductance regulator
CFTR-RD: CFTR-related disease
ECFS: European Cystic Fibrosis Foundation
ENac: Epithelial sodium channel
ICM: Intestinal current measurement
IRT: Immunoreactive trypsinogen
LMICs: Low- and middle-income countries
NBS: Newborn screening
NGS: Next-generation sequencing
NPD: Nasal potential difference
PAP: Pancreas-associated protein
QPIT: Quantitative pilocarpine iontophoresis test
